# Gatekeeper of pluripotency: A common Oct4 transcriptional network operates in mouse eggs and embryonic stem cells

**DOI:** 10.1186/1471-2164-12-345

**Published:** 2011-07-05

**Authors:** Maurizio Zuccotti, Valeria Merico, Michele Bellone, Francesca Mulas, Lucia Sacchi, Paola Rebuzzini, Alessandro Prigione, Carlo A Redi, Riccardo Bellazzi, James Adjaye, Silvia Garagna

**Affiliations:** 1 Sezione di Istologia ed Embriologia, Dipartimento di Medicina Sperimentale, Universita' degli Studi di Parma, Parma, Italy; 2 Fondazione I.R.C.C.S. Policlinico San Matteo, Pavia, Italy; 3 Laboratorio di Biologia dello Sviluppo, Dipartimento di Biologia Animale, Universita' degli Studi di Pavia, Pavia, Italy; 4 Centro di Ingegneria Tissutale, Universita' degli Studi di Pavia, Pavia, Italy; 5 Dipartimento di Informatica e Sistemistica, Universita' degli Studi di Pavia, Pavia, Italy; 6 Molecular Embryology and Aging Group, Department of Vertebrate Genomics. Max-Planck Institute for Molecular Genetics, Berlin, Germany; 7 Centro di Eccellenza in Biologia Applicata, Universita' degli Studi di Pavia, Pavia, Italy

## Abstract

**Background:**

Oct4 is a key factor of an expanded transcriptional network (Oct4-TN) that governs pluripotency and self-renewal in embryonic stem cells (ESCs) and in the inner cell mass from which ESCs are derived. A pending question is whether the establishment of the Oct4-TN initiates during oogenesis or after fertilisation. To this regard, recent evidence has shown that Oct4 controls a poorly known Oct4-TN central to the acquisition of the mouse egg developmental competence. The aim of this study was to investigate the identity and extension of this maternal Oct4-TN, as much as whether its presence is circumscribed to the egg or maintained beyond fertilisation.

**Results:**

By comparing the genome-wide transcriptional profile of developmentally competent eggs that express the OCT4 protein to that of developmentally incompetent eggs in which OCT4 is down-regulated, we unveiled a maternal Oct4-TN of 182 genes. Eighty of these transcripts escape post-fertilisation degradation and represent the maternal Oct4-TN inheritance that is passed on to the 2-cell embryo. Most of these 80 genes are expressed in cancer cells and 37 are notable companions of the Oct4 transcriptome in ESCs.

**Conclusions:**

These results provide, for the first time, a developmental link between eggs, early preimplantation embryos and ESCs, indicating that the molecular signature that characterises the ESCs identity is rooted in oogenesis. Also, they contribute a useful resource to further study the mechanisms of Oct4 function and regulation during the maternal-to-embryo transition and to explore the link between the regulation of pluripotency and the acquisition of de-differentiation in cancer cells.

## Background

The first cell divisions of the preimplantation embryo rely on a number of maternal-effect factors that have been stored in the egg throughout folliculogenesis and that guide early development during the maternal-to-embryo transition, when embryonic genome activation (EGA) occurs and novel transcripts and proteins are produced as a requirement for further development [1]. If the expression of single maternal-effect genes is experimentally altered during mouse oogenesis or in the zygote, most of the embryos arrest development at the 2-cell stage or a few cell divisions later in preimplantation [2]. A question that remains unanswered is concerned with the nature of the transcriptional networks (TN) in which maternal-effect genes operate. This knowledge would further our understanding of the molecular identity of a developmentally competent egg (metaphase II, MII, oocyte) and would allow to investigate how this identity is modified during the switch to an embryonic control of development.

*Oct4 *(Pou5f1, POU domain, class 5, transcription factor 1) is one of the 27 maternal-effect genes reported so far [2] whose transcripts inherited by the zygote are necessary for development beyond the 2-cell stage [3]. Most of our knowledge on Oct4 functions comes from studies that describe its key role in the control of transcriptional regulatory circuits that maintain pluripotency in the inner cell mass (ICM) of the blastocyst [4] and in embryonic stem cells (ESCs) [5-10]. Furthermore, OCT4 is recognised for its capacity, when ectopically expressed in combination with other transcription factors (i.e., NANOG, SOX2, cMYC, KLF4 or ESRRB), to reprogram differentiated cells into pluripotent cells (induced pluripotent stem cells, iPS cells) [11-15].

Recent studies have also shown a role for OCT4 in the acquisition of the egg developmental competence [16,17]. During oocyte growth the OCT4 protein is first detected at the time of follicle recruitment, only in one of two major classes of oocytes present in the mouse ovary, named surrounded nucleolus (SN) oocytes and recognisable for the presence of a ring of heterochromatin surrounding their nucleolus; on the contrary, OCT4 expression is comparativelly down-regulated in NSN (not surrounded nucleolus) oocytes that lack of a ring of heterochromatin around the nucleolus [18-22]. This distinct pattern of expression is maintained throughout oocyte growth, in fully matured antral SN and NSN oocytes and in their derived MII^SN ^and MII^NSN ^oocytes, respectively [17]. The most striking difference between these two categories of oocytes is that only MII^SN ^oocytes may develop beyond the 2-cell stage and reach full term development [23-25]. OCT4 down-regulation in MII^NSN ^oocytes correlates with the down-regulation of the maternal-effect factor STELLA [26] and with the up-regulation of eighteen OCT4-regulated genes that are part of a gene expression network implicated in mitochondrial dysfunction and apoptosis [16], explaining the developmental block encountered by 2-cell embryos obtained from MII^NSN ^oocytes (2-cell^NSN^). This data indicate that *Oct4 *is an important component of a maternal regulatory TN that influences positively (when *Oct4 *is expressed) or negatively (when *Oct4 *is down-regulated) the oocyte developmental competence. The molecular identity and extension of this TN, as much as whether its presence is circumscribed to the egg or, after fertilisation, is maintained beyond the first mitotic division, remains to be understood.

In the present study, by comparing the genome-wide transcriptional profile of ovulated MII oocytes that express the OCT4 protein (MII control, MII^ctrl^) to that of MII oocytes in which OCT4 is comparativelly down-regulated (MII^NSN^), we unveiled an expanded maternal Oct4-TN made of 182 genes. Then, by comparing the transcriptional profile of 2-cell embryos derived from MII^ctrl ^oocytes (2-cell^ctrl^) to that of embryos derived from MII^NSN ^oocytes (2-cell^NSN^), we showed that the Oct4-TN has a core group of 80 genes that remains expressed beyond fertilisation and the first segmentation division. Of these 80 genes, 37 are notable companions of the Oct4 transcriptome in ESCs and the majority is expressed in cancer cells.

## Results

### Gene expression profiles of developmentally incompetent and competent MII oocytes or 2-cell embryos

To highlight genes with altered expression (up- or down-regulated) in developmentally incompetent MII^NSN ^oocytes, we first compared their transcription profile with that of MII^ctrl ^oocytes using microarray data from our previous work [17]. The data lists obtained earlier were revised since the data banks from which information was recovered are constantly updated.

**Figure 1:**
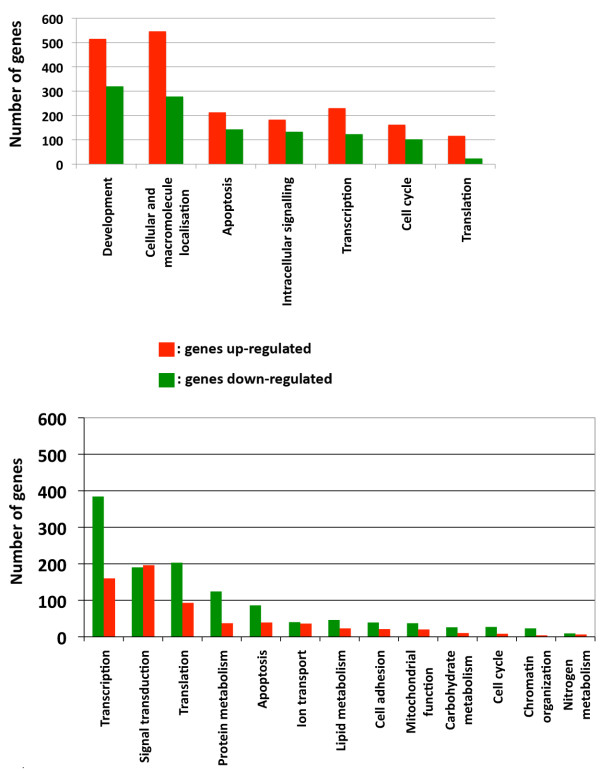
**Microarray-based analysis of the transcription profile of developmentally incompetent and competent MII oocytes and 2-cell embryos**. (A) Major biological processes and functions found when comparing the transcription profile of MII^NSN ^vs. MII^ctrl ^oocytes and number of up- and down-regulated genes in each of these processes. (B) Major biological processes and functions found when comparing the transcription profile of 2-cell^NSN ^vs. 2-cell^ctrl ^embryos and number of up- and down-regulated genes in each of these processes.

A list of regulated and annotated genes or gene sequences (from now on named genes) was retrieved after setting a 1.5 fold-change threshold and a detection p value ≤ 0.01. Using the Gene Ontology (GO) enrichment analysis tool provided by the data mining and bioinformatics software Orange http://www.ailab.si/orange, 3102 (Additional file 1) out of 8354 regulated genes were assigned to seven major biological processes (Figure 1A), including development, cellular and macromolecule localisation, apoptosis, transcription, intracellular signalling, cell cycle and translation. This analysis showed that the great majority of these genes were up-regulated in MII^NSN ^oocytes (Figure 1A).

Next, using the same fold-change and p value thresholds, we generated another list of regulated genes by comparing the transcription profile of 2-cell^NSN ^vs. 2-cell^ctrl ^embryos. Out of 3599 regulated genes, 1887 (Additional file 2) were assigned to thirteen major biological processes. Figure 1B shows the number of up- and down-regulated genes in each of these processes.

In summary, we retrieved two lists of regulated genes that highlight the changes occurring to the transcriptional signature of developmentally competent eggs or 2-cell embryos, when compared to their incompetent counterparts. Our next step was aimed at the identification of known Oct4-regulated genes within each of these two lists.

### A maternal Oct4 transcriptional network is constituent of the molecular identity of both MII oocytes and 2-cell embryos

**Figure 2:**
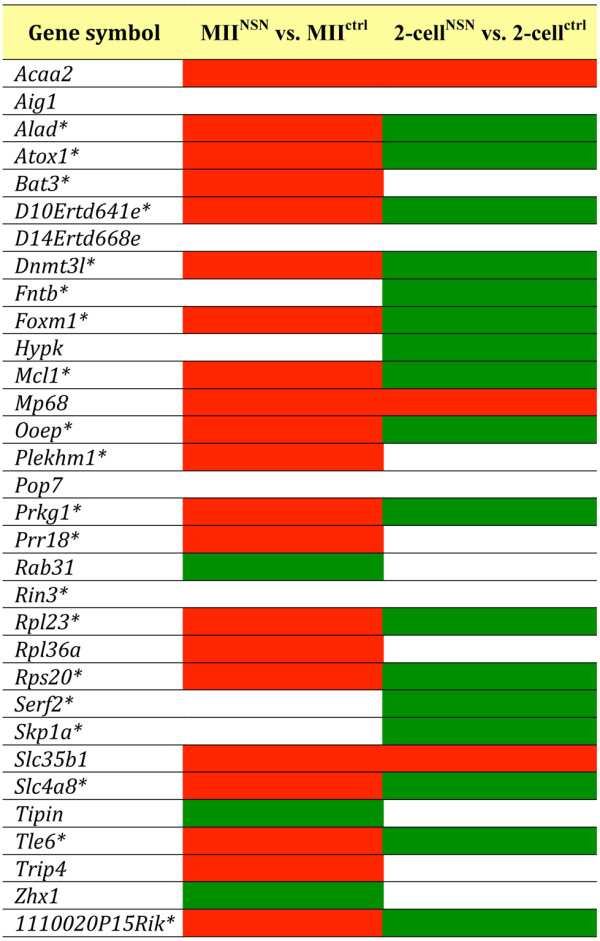
**List of OCT-4-regulated genes expressed in both MII oocytes and 2-cell embryos**. Comparison of gene expression is made between MII^NSN ^vs. MII^ctrl ^or 2-cell^NSN ^vs. 2-cell^ctrl^. Red box, up-regulated; green box, down-regulated; blank box, not differentially expressed. The hypergeometric test proved that the up- (MII^NSN ^oocytes) and down-regulated (2-cell^NSN ^embryos) pattern of expression of the majority genes (*) was not a stochastic event (p = 0.0039).

Using mouse and human chip datasets of OCT4-regulated genes in ESCs [5,27,28], we singled out a group of 32 OCT4-regulated genes whose transcripts were detected in both the MII oocyte and 2-cell embryo microarray lists. When compared to MII^ctrl ^samples, the great majority of these genes were up-regulated in developmentally incompetent MII^NSN ^oocytes in which the OCT4 protein is markedly down-regulated (Figure 2), suggesting a down-regulatory function of this transcription factor over these genes. By comparing 2-cell^NSN ^with 2-cell^ctrl ^embryos, we found that the expression of the majority of this group of 32 genes was higher in the latter (Figure 2), indicating that the down-regulatory function of OCT4 had been released. In fact, following fertilisation, the maternal OCT4 protein present in MII^ctrl ^oocytes is carried over into the zygote and by the 2-cell stage becomes undetectable [29], to reappear again, expressed from the embryonic genome, at the 8-cell stage [30].

The hypergeometric test confirmed that the up- and down-regulated pattern of expression of 20 of these OCT4-regulated genes in MII^NSN ^oocytes and 2-cell^NSN ^embryos (Figure 2), respectively, was not a stochastic event, but instead a specific characteristic of this group of genes at these two developmental stages (p = 0.0039). The results of the microarray analysis for five of these genes were confirmed by qRT-PCR (Additional file 3).

**Figure 3:**
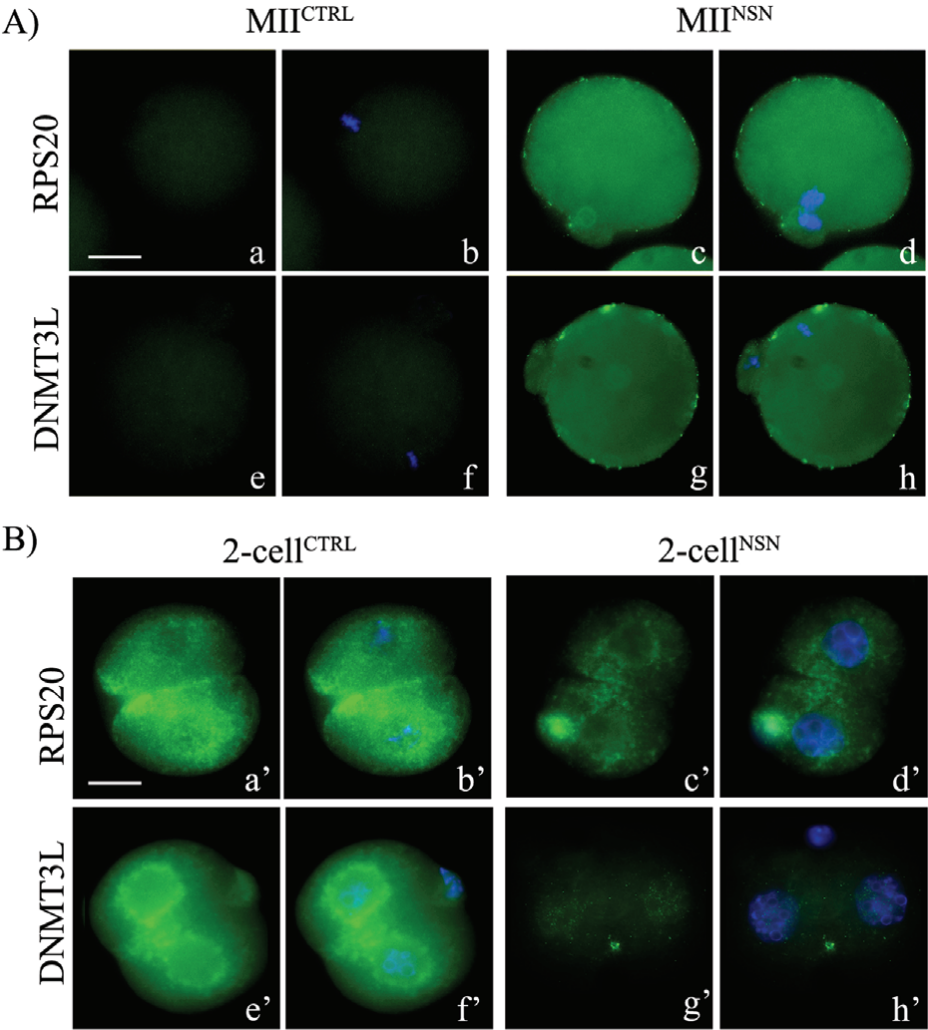
**Immuno detection of the RPS20 and DNMT3L proteins in MII oocytes and 2-cell embryos**. (A) MII^ctrl ^and MII^NSN ^oocytes and in (B) 2-cell^ctrl ^and 2-cell^NSN ^embryos. (a, c, e, g and a', c', e', g'), antibodies staining; (b, d, f, h and b', d', f', h'), merge between antibodies staining and DAPI counterstaining. Scale bar, 20 μm.

Of these 20 OCT4-regulated genes, we analysed the expression profile of those proteins for which an antibody was commercially available, i.e., DNMT3L1, RPS20 and MCL1 (MCL1 antibody did not give consistent results and therefore was not used further, data not shown). DNMT3L is a crucial factor for the establishment of genomic imprinting in oocytes and the expression of *Dnmt3l *increases during preimplantation in both mouse and rhesus monkey, suggesting a developmental role [31]. RPS20 is a ribosomal protein involved in translation and its role in preimplantation as never been investigated before. Immunolabeling of DNMT3L and RPS20 antibodies was positive in MII^NSN ^oocytes (Figure 3A) and 2-cell^ctrl ^(Figure 3B), whereas it was negative in MII^ctrl ^(Figure 3A) and 2-cell^NSN ^embryos (Figure 3B), confirming the reversal pattern of expression described for their transcripts during the passage from the egg to the 2-cell stage.

Our next step was aimed at determining whether the Oct4-TN could be further expanded and better characterised.

### Numerous genes of the maternal Oct4 transcriptional network are known members of the Oct4 interactome in ESCs

**Figure 4:**
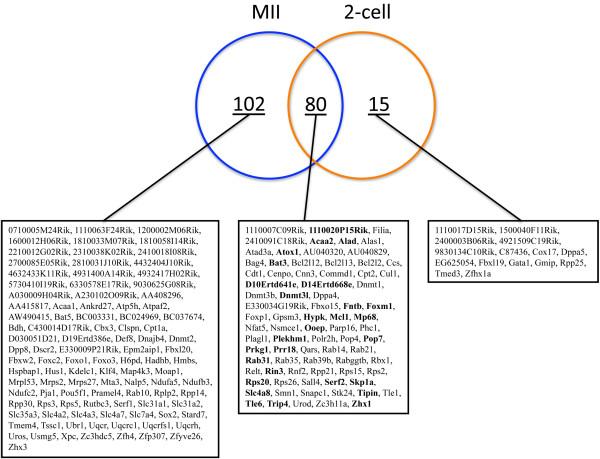
**Venn diagram illustrating distinct and overlapping gene expression patterns in MII oocytes and 2-cell embryos**. Boxes, list of genes belonging to each specific group; genes in bold, OCT4-regulated genes.

**Figure 5:**
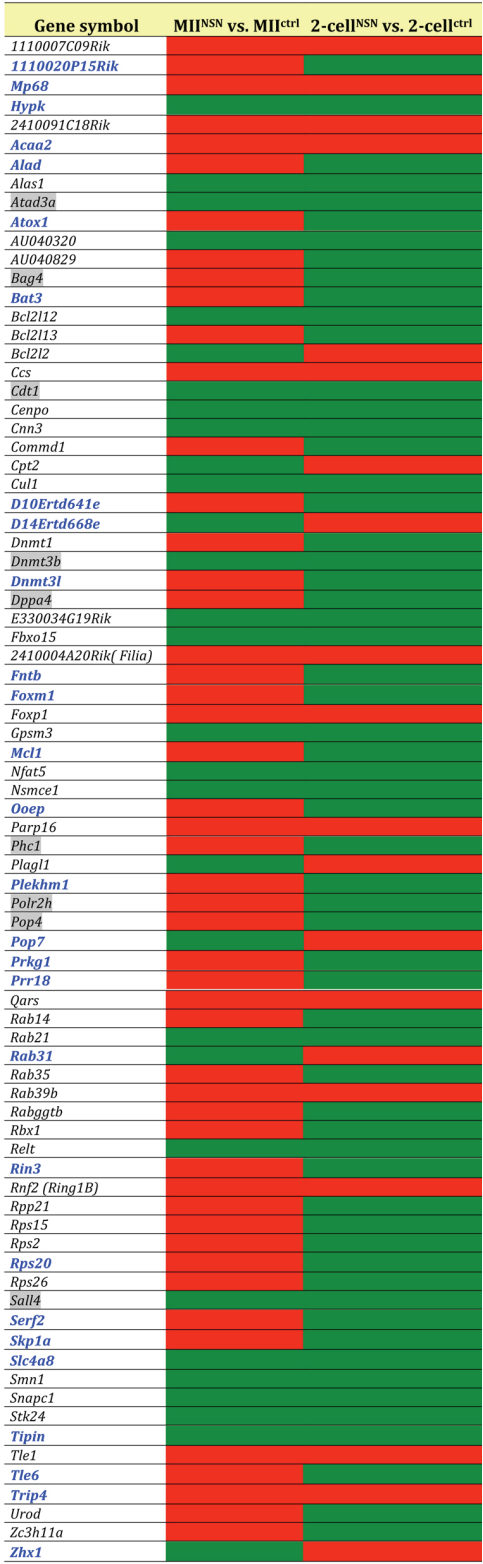
**Oct4 transcriptional network**. Genes expressed in both MII oocytes and 2-cell embryos. Green box, down-regulated; red box, up-regulated; blue font, OCT4-regulated gene; highlighted in grey, OCT4-correlated gene (Campbell et al., 2007).

Using the Network Explorer module provided by the Orange software (see Methods for details), we explored public databases for links (based on GO and MeSH, Medical Subject Headings, terms) between the group of 32 OCT4-regulated genes used as bait (with the addition of *Oct4*), and all the annotated mouse gene sequences. This search retrieved an annotation network made of a total of 312 genes (Additional file 4), 197 of which were components (i.e., expressed) of our MII oocyte and/or 2-cell embryo list of regulated genes. This network was combined with the results of gene expression differential analysis to infer transcriptional relationships among the genes of an expanded Oct4-TN. The expanded Oct4-TN, made of 197 genes (Figure 4), comprised 102 genes expressed exclusively in MII oocytes (Additional file 5), 15 genes solely in 2-cell embryos (Additional file 6) and 80 genes in both MII oocytes and 2-cell embryos (from now onwards named Oct4-OETN, Oocyte-to-Embryo Transcriptional Network; Figure 5). The Oct4-OETN contained all the 32 OCT4-regulated genes, except 4 that were not annotated and thus excluded; most (20) of the remaining 28 genes were up-regulated in MII^NSN ^oocytes but down-regulated in 2-cell^NSN ^embryos. Besides these 28 OCT4-regulated genes, the Oct4-OETN included 8 more genes of a recently published list of OCT4-correlated transcripts expressed in ESCs [32] (Figure 5) and 44 genes for which a direct or indirect action of OCT4 on their expression will need to be further investigated.

When compared to their respective control samples, more than half of the Oct4-OETN genes (51 out of 80) were up-regulated in MII^NSN ^oocytes but down-regulated (59 genes) in 2-cell^NSN ^embryos; 7 genes were down-regulated in oocytes and up-regulated in embryos, whereas 22 and 14 genes where down- and up-regulated, respectively, in both oocytes and embryos (Figure 5).

Among the 15 newly expressed genes, *Dppa5 *(Developmental pluripotency associated gene 5), *Gata1 *and *Zeb1 *are the best known and their main functions will be described in the section below.

In summary, this analysis brought to light in MII oocytes, a maternal Oct4-TN made of 182 genes. Within this circuitry, we could identify a restricted Oct4-OETN made of 80 genes as core component common to the molecular identity of both eggs and 2-cell embryos. Almost half (37] of the Oct4-OETN genes are known Oct4 companions in ESCs, as their expression is directly regulated by [5,27,28] or correlated with [32]*Oct4*. Our next step was aimed at investigating the main functional characteristics of the genes and gene networks of the expanded Oct4-TN.

### Dissecting the expanded Oct4 transcriptional network identifies nineteen gene clusters

**Figure 6:**
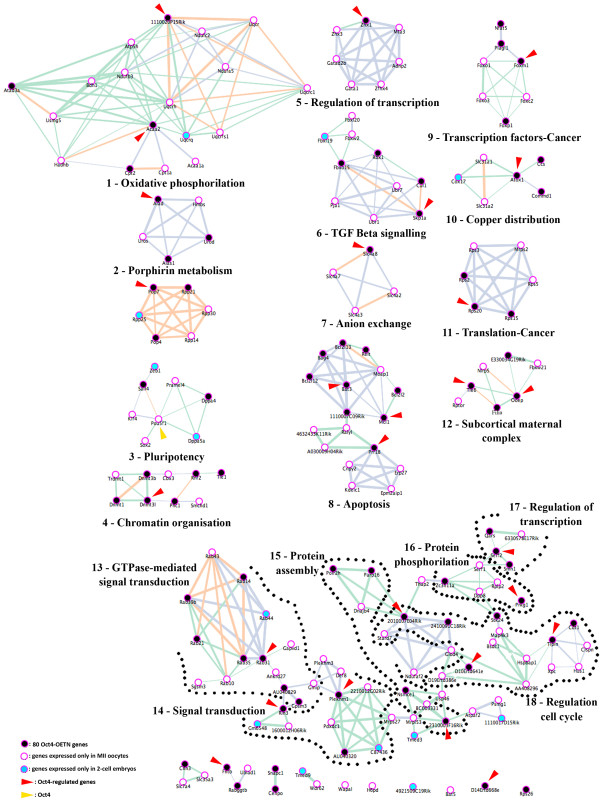
**Gene clusters singled out in the expanded Oct4-TN**. Green lines, MeSH annotations; orange lines, MeSH and GO annotations; grey lines, GO annotations. Increasing line width indicates stronger annotation relationship.

The Oct4-TN was further analysed with the Network Explorer module limiting to < 3 the number of sequential connections between one of the 28 OCT4-regulated genes and the annotated neighbours. We identified 19 distinct gene clusters (Figure 6), each containing at least one of the 80 Oct4-OETN genes. Based on GO annotations and on a literature catalogues search, 18 of the 19 clusters could be ascribed to a major biological function. A description of the main characteristics of each gene cluster and of those Oct4-OETN genes for which functional details were retrieved is given in Additional file 7[33-92]. In summary, those Oct4-OETN genes for which we could retrieve solid information fell into three main categories with > 3 genes: 1) cancer, 18 genes (i.e., *Rab39b*, *Rab35*, *Rab31*, *Rab21*, *Rab14*, *Rps15*, *Rps20*, *Rps2*, *Atox1*, *Plagl1*/*Zac1*, *Foxp1*, *Foxm1*, *Nfat5*, *Cdt1*, *Ring1B*, *Phc1*, *Tle1 *and *Atad3a*); 2) preimplantation development-pluripotency, 14 genes (i.e., *Dppa4*, *Sall4*, *Dnmt3b*, *Dnmt1*, *Dnmt3l*, *Dnmt3a*, *Ring1B*, *Scl4a8*, *Plagl1*/*Zac1*, *Zhx1*, *Commd1*/*U2af1*-*rs1*, *Ooep*, *Filia *and *Tle6*); 3) cell division, 4 genes (i.e., *Gpsm3*, *Cdt1*, *Skp1a *and *Tipin*). Among the poorly known genes remaining, there is a group made of 7 genes (*Bag4*, *Bat3*, *Bcl2l12*, *Bcl2l13*, *Bcl2l2*, *Mcl1 *and *Relt*) with apoptotic/anti-apoptotic functions, whereas all the others could not be grouped as they fell into several different categories, each with less than three genes. This information improves our understanding of the maternal Oct4-TN composition, but also will serve as basic knowledge for further dissection and future studies of its role in oogenesis and preimplantation development.

### Most genes of the maternal Oct4 transcriptional network are also expressed in cancer cells

Since one of the most abundant categories singled out when dissecting the expanded Oct4-TN correlated with cancer, we interrogated a more specific repository of cancer-related genes, i.e., genes that, compared to controls, are significantly up- or down-regulated in a wide variety of solid and non-solid tumours (EBI Gene Expression Atlas; http://www.ebi.ac.uk/gxa/). Strikingly, the great majority, 157 out of 197 (79,7%) (Additional file 8) of the expanded Oct4-TN and 65 out of 80 Oct4-OETN genes (81.2%) (Additional file 9), were recognised as cancer-related genes. The non-stochastic nature of these frequencies was confirmed by the hypergeometric test (p = 0.0031).

## Discussion

**Figure 7:**
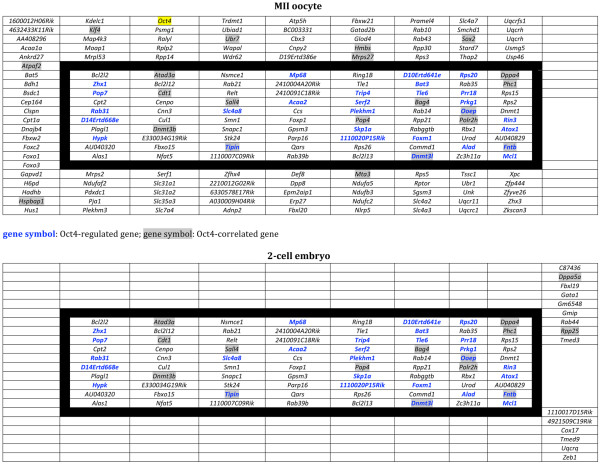
**The Oct4-TN genes expressed in MII oocytes and in 2-cell embryos**. Black frame, 80 Oct4-OETN genes expressed in both MII oocytes and 2-cell embryos; highlighted in yellow, *Oct4*.

Each cell type in our body has its own molecular identity defined by a number of transcriptional networks that operate and cooperate to maintain the cell integrity and a specific undifferentiated/differentiated status. During cell differentiation some transcriptional network die out or fade one into another while guiding the cell towards the acquisition of a specific phenotype. Transcriptional inheritance is the load of transcripts and active genes that are passed to the subsequent step of differentiation. Likewise, the mammalian egg reaches the fertilisation encounter with a transcriptional inheritance representative of its developmental legacy. As part of this molecular identity, in this study we brought to light an Oct4-TN of maternal origin that is present during the developmental period comprised between the MII oocyte and the 2-cell embryo (Figure 7). As hereafter described, these results allowed the generation of novel hypotheses on the developmental role of a maternal Oct4 transcriptional inheritance during the early stages of mouse preimplantation development.

One marked phenomenon that occurs during the developmental interval comprised between ovulation and EGA is the inactivation or degradation of a considerable number of transcripts mainly by processes of deadenylation [93], but also through the association with RNA-binding proteins [94,95] and elimination by small silencing RNAs that degrade mRNAs or repress their translation [96]. The maternal Oct4-TN that we identified has its maximum expansion in MII oocytes, comprising 182 genes, then, following fertilisation, more than half (102) of these transcripts are markedly down-regulated, to become almost undetectable in 2-cell embryos, suggesting their prompt degradation or deadenylation at the beginning of development (our microarrays data were obtained following oligo-dT retrotranscription that amplified only polyadenylated transcripts, see M&M). Interestingly, this group of genes includes *Oct4*, *Sox2 *(whose marked down-regulation in 2-cell^ctrl ^embryos has already been described before) [97,98] and *Klf4*. *Oct4*, *Sox2 *and *Klf4 *are central to the maintenance and promotion of cell pluripotency [99]. Their down-regulation after fertilisation may signal the execution of the egg developmental programme (perhaps carried out by those Oct4-OETN genes that survive degradation, see below), then, at later stages of development, they are re-expressed, but only in some blastomeres, namely those that will contribute to the ICM, to induce their pluripotent status; on the contrary, they are kept down-regulated in those cells that will contribute to the trophectoderm. In support of this hypothesis, a recent paper has demonstrated that *Oct4 *re-expression occurs at the 8-cell stage embryo [30] and is dissimilar in single blastomeres [100], suggesting a possible different developmental commitment. The developmental block encountered by 2-cell^NSN ^embryos could be associated (besides the up-regulation of apoptosis-related genes, as reported before [16,17]), to abnormal expression or distribution of transcripts or proteins following the first segmentation [101]. To this regard, our analysis of DNMT3L and RPS20, whilst demonstrating a differential expression of both transcripts and proteins in 2-cell^ctrl ^vs. 2-cell^NSN ^embryos, it did not evidence a differential distribution in the two blastomeres (Figure 3), although, at this stage, we cannot role out this hypothesis because of the low sensitivity power of an immunocytochemistry analysis.

**Figure 8:**
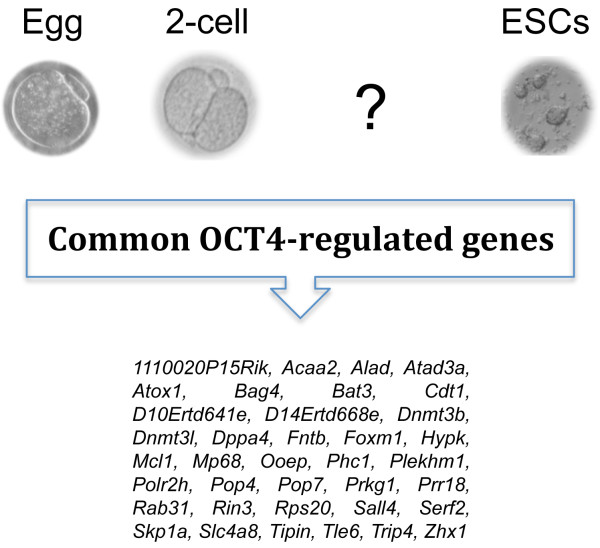
**OCT4-regulated genes expressed in eggs, 2-cell embryos and ESCs**. This core Oct4-TN may provide a link between eggs, early preimplantation embryos and ESCs.

The 80 Oct4-OETN gene transcripts that survive the massive post-fertilisation degradation represent the maternal Oct4-TN inheritance that is passed from the MII oocyte to the 2-cell embryo. Following fertilisation some of the transcripts of these genes might be translated (as shown for the up-regulation of DNMT3L and RPS20) and their proteins, together with those of the group of 15 newly activated genes, may play a role during the following stages of development. This core Oct4-TN, that shares 37 genes with an OCT4 regulatory network active in ESCs, might represent the molecular signature of maternal origin on which the ESCs molecular identity is built up and tailored, thus providing a link between eggs, early preimplantation embryos and ESCs (Figure 8).

The expression of the OCT4 protein during oogenesis first occurs at the time of follicle recruitment [17], when oocytes have reached a size of approximately 30-40 μm in diameter, suggesting that the beginning of the Oct4-TN establishment might occur at this stage of oocyte growth.

The significant presence of cancer-associated genes as part of the Oct4 transcriptome is a theme shared with ESCs [10,102], suggesting that an Oct4 circuitry may be operating also in cancer cells and providing a molecular link between the regulation of pluripotency and the acquisition of dedifferentiation in cancer cells [103-107]. Furthermore, in view of the cancer stem cell hypothesis [108], the presence of an Oct4-TN in cancer cells may help the identification and characterisation of the stem cell population within the tumor.

## Conclusions

In this study we identified an Oct4-TN that is established during oogenesis and that partially survives the wide transcriptional erasure that occurs soon after fertilisation. Its core Oct4-OETN circuitry of 80 genes is maintained up to the 2-cell stage of development and may represent part of the transcriptional signature that is conveyed to the ICM. The Oct4-TN that we described provides a useful resource to 1) further study the mechanisms of Oct4 function and regulation during the maternal-to-embryo transition; 2) explore the link between the regulation of pluripotency and the acquisition of dedifferentiation in cancer cells; 3) improve our understanding of the molecular factors that contribute to the mammalian egg developmental competence and give opportunities for testing new prognostic molecular markers of oocyte quality in animal and human assisted reproduction.

## Methods

### Oocytes isolation, culture to the MII stage and to the 2-cell embryo

Research on mice has been performed after the approval of the Animal Ethics Committee of the University of Pavia. Animals were maintained according to the Guide for Care and Use of Laboratory Animals. Fully matured antral oocytes were isolated from the ovaries of 4-6 week-old B6C3F1 female mice (Charles River, Come, Italy) injected with 3.5 I.U. PMSG (Folligon, Intervet Srl, Italy) and those that had an NSN type of chromatin organisation [16] were cultured to the MII stage [17]. MII^NSN ^and MII^ctrl ^oocytes were inseminated with sperm isolated from the epidydymes of 5 month-old B6C3F1 male mice [24] and those that reached the 2-cell stage, 26 hr after insemination, were further treated for microarray or qRT-PCR analyses.

### Microarray-based global gene expression analysis

Total messenger RNA (mRNA) was isolated using the RNeasy mini kit (Qiagen, USA) and quality-checked by Nanodrop analysis (Nanodrop Technologies, Wilmington, DE, USA). 400ng of mRNA was used as input for generating biotin-labelled cRNA. Two rounds of mRNA amplification were performed using the Illumina TotalPrep RNA Amplification Kit (Ambion, Austin, TX, United States), which is a complete system for generating biotinylated, amplified RNA for hybridisation with Illumina Sentrix arrays. cRNA samples were then hybridised onto Illumina mouse-8 BeadChips version 3. Hybridizations, washing, Cy3-streptavidin staining and scanning were performed on the Illumina BeadStation 500 platform (Illumina, San Diego, CA, USA), according to the manufacturer's instruction. The following samples were hybridised: one 2-cell^ctrl ^and two 2-cell^NSN ^(2-cell^NSN^-a and 2-cell^NSN^-b). Expression data analysis was carried out using the BeadStudio software 3.0 (Illumina, San Diego, CA, USA). The raw microarrays data have been deposited in Gene Expression Omnibus (GEO) with the following GEO accession number, GSE28704.

### Bioinformatic analysis

Raw data were background-subtracted, normalized using the "rank invariant" algorithm and filtered for significant expression on the basis of negative control beads. Genes were considered significantly expressed with detection p values ≤ 0.01. Differential expression analysis was performed with a fold change threshold of 1.5 (Additional file 10).

GO enrichment analysis, file management, network generation and other statistical analysis were performed with Python scripts that integrates several functions provided by the Bioinformatics extension of the Orange Data Mining Suite http://www.ailab.si/orange/.

The enriched GO biological terms were determined using the entire mouse genome as a reference set. A threshold of 0.01 on the enrichment p values was set as a measure of statistical significance. The enriched GO processes were further automatically classified into a set of macro categories defined by the domain experts.

The annotation network that was used to infer transcriptional relationships within the Oct4-TN was generated through a literature-based search strategy. This methodology retrieved all the PubMed publications related to the genes in the mouse genome and assigned to each gene a set of MeSH and GO annotation terms. A text-mining method based on the annotation terms was used to calculate the similarity between genes [109]. For each pair of genes in the TN, a connecting link was created if the annotation similarity exceeded a cut-off value of 0.7.

Cancer-related genes were identified from experiments in EBI Atlas database by setting a p value threshold of 0.05.

### Real-time polymerase chain reaction

Total RNA was extracted separately from 10 embryos in 3 μl of Lysis Buffer [110]. Retrotranscription was performed in a 20 μl reaction mixture containing: 3 μl of RNA, 1× PCR buffer, 5 mM MgCl_2_, 4 mM of each dNTP, 0.625 μM oligo d(T)_16_, 1.875 μM Random Hexamers, 20 U RNase Inhibitor, 50 U MuLV reverse transcriptase (Applera). The reverse transcription was performed at 25°C for 10 min, 42°C for 60 min, 99°C for 5 min. A mixture of the cDNA products from the 10 embryos was generated and one twentieth of the resulting cDNA was amplified in duplicate by Real-Time PCR in 20 μl reaction mixture with 200 nM of each specific primer (Additional file 11) and the MESA GREEN qPCR MasterMix Plus for SYBR assay no ROX sample (Eurogentec) at 1× as final concentration. The amplification reaction was performed in a Rotorgene 6000 (Corbett Life Science) as follows: 95°C for 5 min, followed by 40 cycles at 95°C for 10 sec, 60°C for 15 sec, 72°C for 20 sec. The Rotorgene 6000 Series Software 1.7 was used for the comparative concentration analysis. *Htatsf1 *gene expression was used for the normalisation of the samples.

### Immunofluorescence analysis

Oocytes and embryos were fixed with freshly prepared 4% paraformaldehyde for 20 min, permeabilised with 0.5% Triton X-100 for 20 min at 4°C and treated with 0.5% blocking reagent (Roche, Boston, MA) in TNT (0.1 M, Tris-HCl, pH 7.5, 0.15 M NaCl, 0.05% Tween-20) buffer for 20 min at 4°C. Immunostaining was performed with rabbit anti-RPS20 polyclonal antibody (Abcam; ab74700, 1:2000), rabbit anti-DNMT3L polyclonal antibody (Abcam; ab3493, 1:500) or rabbit anti-MCL1 monoclonal antibody (Abcam; ab32087, 1:20) for 1 h at 37°C. Primary antibodies were detected using a secondary Alexa Fluor488-goat anti-rabbit IgG (Molecular Probes; 1:400 diluted in PBT: 1× PBS plus 0.1% Tween 20) antibody for 1 h at 37°C. Samples were then washed in PBT (three times) for 15 min at 4°C, counterstained with DAPI (0.2 μg/ml in PBS for 5 min) and mounted in Vectashield (Vector).

## Competing interests

The authors declare that they have no competing interests.

## Authors' contributions

MZ conceived the study, participated in its design and coordination, participated in the bioinformatic analyses and worked on drafting the manuscript; VM did the immunocytochemistry analyses; MB isolated the embryos; LS, FM and RB did the bioinformatic analyses; PR, did the RT-PCR analyses; AP, did the microarrays analysis; CAR worked on the elaboration of the study; JA conceived the study, participated in its design and coordination, did the microarrays analyses and worked on drafting the manuscript; SG conceived the study, participated in its design and coordination and worked on drafting the manuscript. All authors read and approved the final manuscript.

## Supplementary Material

Additional file 1 **GO enrichment**. List of regulated genes (MII^NSN ^vs. MII^ctrl^). Click here for file

Additional file 2 **GO enrichment**. List of regulated genes (2-cell^NSN ^vs. 2-cell^ctrl^) Click here for file

Additional file 3 **qRT-PCR expression profile of *Oct4 *and five Oct4-regulated genes**. This analysis confirmed the down-regulated pattern of expression detected by microarray analysis when comparing 2-cell^NSN ^vs. 2-cell^ctrl ^embryos. Click here for file

Additional file 4 **Gene annotation similarity network**. Gene annotation similarity network made of 312 genes retrieved when exploring the public databases for GO and MeSH links between the group of 32 Oct4-regulated genes and all the annotated mouse gene sequences. Green lines, MeSH annotations; orange lines, MeSH and GO annotations; grey lines, GO annotations. Increasing line width indicates stronger annotation relationship. Red dot, *Oct4 *gene. Click here for file

Additional file 5 **Oct4 transcriptional network in MII oocytes**. Genes expressed exclusivelly in MII oocytes. Green box, down-regulated; red box, up-regulated. Click here for file

Additional file 6 **Oct4 transcriptional network in 2-cell embryos**. Genes expressed exclusivelly in 2-cell embryos. Green box, down-regulated; red box, up-regulated. Click here for file

Additional file 7 **Main characteristics of the Oct4-OETN genes**. Main characteristics of the genes found in each of the 18 gene clusters. Click here for file

Additional file 8 **List of cancer-related genes present in the expanded Oct4-TN**. The majority of the genes belonging to the expanded Oct4-TN were recognised as cancer-related genes. Click here for file

Additionalfile 9 **List of cancer-related genes present in the Oct4-OETN**. The majority of the genes belonging to the Oct4-OETN were recognised as cancer-related genes. Click here for file

Additional file 10 **Microarray analysis**. Microarray analysis of 2-cell embryos. Click here for file

Additional file 11 **RT-PCR primers**. List of primers used for the real time RT-PCR analysis. Click here for file
