# Supplementary figures and images for: Gatekeeper of pluripotency: A common Oct4 transcriptional network operates in mouse eggs and embryonic stem cells
Source: BMC Genomics. 2011 Jul 5;12:345. doi: 10.1186/1471-2164-12-345 (PMC3154874; doi:10.1186/1471-2164-12-345)

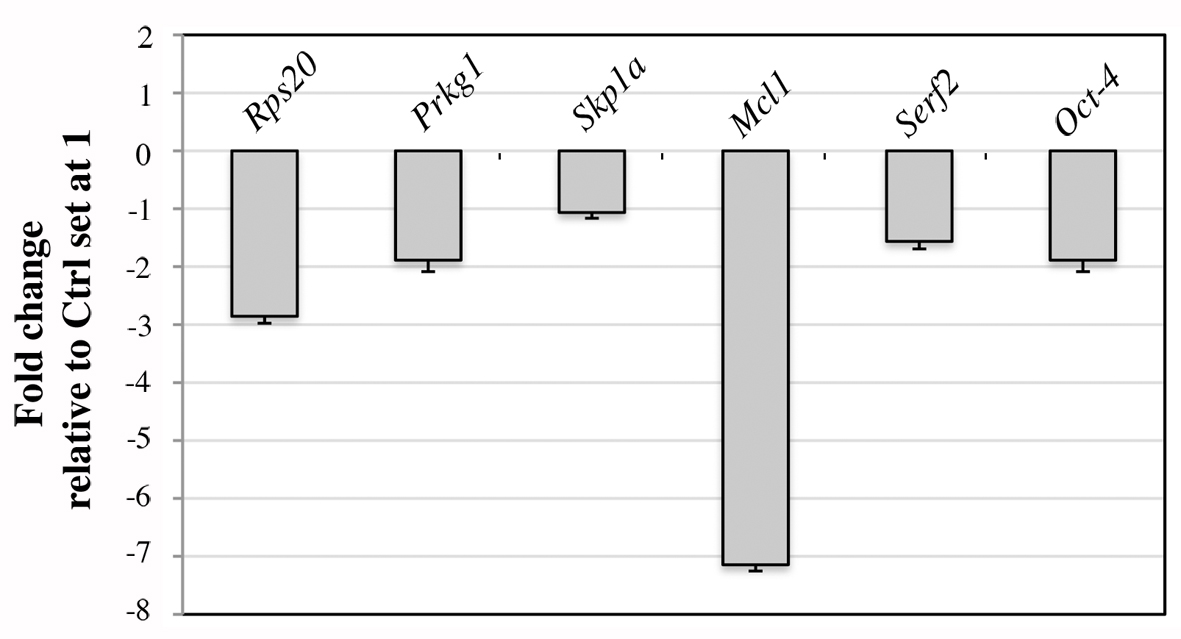

Supplement: Additional file 3 — qRT-PCR expression profile of Oct4 and five Oct4-regulated genes. This analysis confirmed the down-regulated pattern of expression detected by microarray analysis when comparing 2-cellNSN vs. 2-cellctrl embryos. [file 1471-2164-12-345-S3.JPEG]

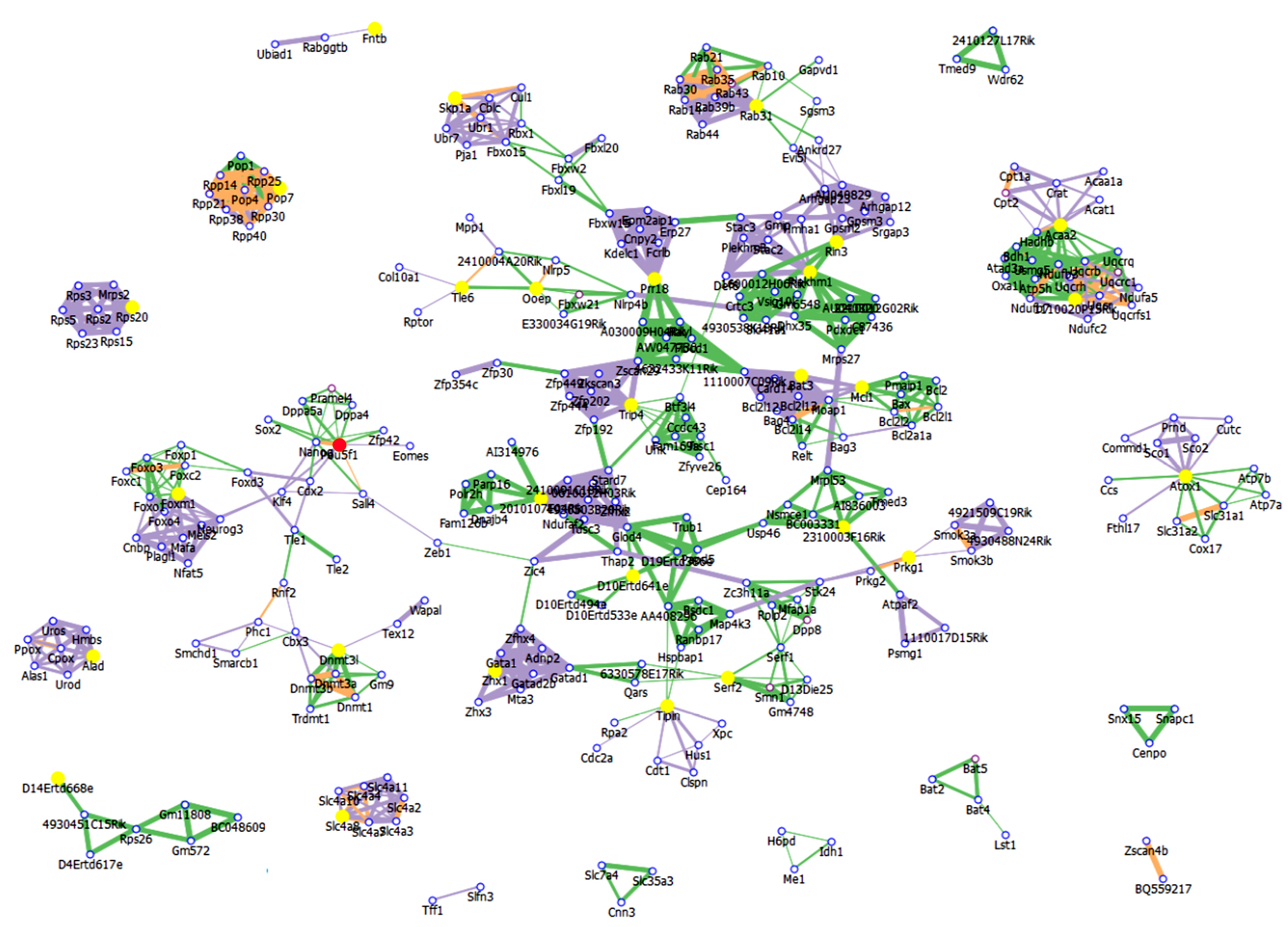

Supplement: Additional file 4 — Gene annotation similarity network. Gene annotation similarity network made of 312 genes retrieved when exploring the public databases for GO and MeSH links between the group of 32 Oct4-regulated genes and all the annotated mouse gene sequences. Green lines, MeSH annotations; orange lines, MeSH and GO annotations; grey lines, GO annotations. Increasing line width indicates stronger annotation relationship. Red dot, Oct4 gene. [file 1471-2164-12-345-S4.JPEG]
