# Supplementary material for: Gatekeeper of pluripotency: A common Oct4 transcriptional network operates in mouse eggs and embryonic stem cells
Source: BMC Genomics. 2011 Jul 5;12:345. doi: 10.1186/1471-2164-12-345 (PMC3154874; doi:10.1186/1471-2164-12-345)
Supplement: Additional file 7 — Main characteristics of the Oct4-OETN genes. Main characteristics of the genes found in each of the 18 gene clusters. [file 1471-2164-12-345-S7.DOC]

**Additional file 7.** Main characteristics of the Oct4-OETN genes found in each of the 18 gene clusters.

Eleven Oct4-OETN genes (*1110007C09Rik, 1110020P15Rik, 2010107E04Rik, 2310003F16Rik, 2410004A20Rik, 2410091C18Rik, AU040320, AU040829, D10Ertd641e, D14Ertd668e* and *E330034G19Rik*), though included in the clusters, were not provided with clear GO annotations and could not be assigned to a specific function.

*Gene cluster 1*, ‘Oxidative phosphorilation’. Most of the genes of this cluster encode for mitochondrial proteins involved in oxidative phosphorilation (Nduf family) or fatty acid metabolism (*Cpt1a*, *Cpt2* and *Acaa2*). It includes three (*Atad3a*, *Cpt2* and *Acaa2*) OET genes. *Atad3a*, a member of the Atpase family which function is poorly known, although it has been identified as a possible novel target molecule in malignant cells [32].

*Gene cluster 2*, ‘Porphirin metabolism’. Contains three (*Alad*, *Urod* and *Alas1*) Oct4-OETN genes. *Alad* and *Urod* are involved in the heme biosynthetic process. *Alas1* is the ubiquitous form of the 5-aminolevulinate synthase 2 gene, crucial for erythropoiesis.

*Gene cluster 3*, ‘Pluripotency’. Most of these genes are associated with cell pluripotency. Besides *Oct4*, it includes three Oct4-OETN genes (*Dppa4*, *Sall4* and *Tle1*). *Dppa4* (developmental pluripotency-associated gene 4) encodes for a putative SAP domain protein expressed in pluripotent stem cells, in embryonal carcinoma cells, in primordial germ cells, in oocytes throughout folliculogenesis, during preimplantation development and then it is restricted to the ICM at the blastocyst stage [33]. A recent study has demonstrated that DPPA4 is dispensable for ESCs maintenance of pluripotency as well as for germ cells differentiation, but it seems important in postimplantation development [34]. DPPA4 and its companion DPPA2 play a role during the early stages of development, as evidenced by their marked down-regulation in blocked 2-cell embryos obtained following injection of the mRNA of the mutant *Zar1L* gene at the zygote stage [35]. SALL4, a transcription factor of the spalt family, is a zinc finger modulator of the level of *Oct4* expression, it is crucial in the maintenance of ESCs pluripotency [36], in the formation of the epiblast and primitive endoderm from the ICM of the blastocyst [37]. *Sall4* and *Oct4* are part of a transcriptional feedback network in ESCs whereby *Sall4* is a master regulator that works antagonistically to *Oct4* [38]. Both *Sall4* and *Oct4* are expressed early in oogenesis [39, 40, 17]. *Tle1* (Transducin-Like Enhancer 1) is a member of the groucho/TLE family of genes that interacts with the transcription factor RUNX2 to function as a transcriptional co-repressor binding to rRNA genes during mitosis and interphase. Down-regulation of *Tle1* alleviates RUNX2-mediated repression of rRNA genes transcription and selectively increases histone modifications linked to active transcription. Loss of TLE-dependent rRNA gene regulation induces global protein synthesis and enhances cell proliferation [41]. Its over-expression is used as a diagnostic marker for synovial sarcomas [42]. Its function during oogenesis and preimplantation development is unknown.

The ‘pluripotency’ cluster includes also *Dppa5* (known also as *Esg1*, embryonic stem cell-specific gene 1) and *Zeb1*, two of the 15 genes of the expanded Oct4-TN that are expressed exclusively in 2-cell embryos. The up-regulation of *Dppa5* that we found confirms the pattern of expression in eggs and early preimplantation embryos that had been shown before by RT-PCR [43]. Whilst early studies suggested a role of *Dppa5* in ESCs pluripotency [44], more recently, its targeted disruption showed that the gene is dispensable for their self-renewal and establishment of germ cells [45]. *Zeb1* has been shown to have a role in the promotion of tumours [46, 47].

*Gene cluster 4*, ‘Chromatin organisation’. This cluster contains five *(Dnmt1, Dnmt3b, Dnmt3l, Phc1* and *Rnf2/Ring1B*) Oct4-OETN genes. DNA methyltransferases are enzymes involved in the regulation of CpG methylation. DNMT1 is involved in the maintenance of methylation imprints during preimplantation development, whereas DNMT3a and DNMT3b are *de novo* methyltransferases involved in the establishment of methylation imprints during gametogenesis [48]. Whilst DNMT3a and DNMT3b are dispensable, DNMT1 is sufficient to maintain the imprinted methylation signatures [49, 50]. All these enzymes are expressed in ESCs, where DNMT3a and DNMT3b may also contribute to the maintenance, beside the establishment of methylation profiles [51]. The *Dnmt3l* gene has three promoters: the most 5’ is active in oocytes, a second is active in prospermatogonia and ESCs and a third promoter is active in late pachytene spermatocytes [52]. DNMT3L is important for the establishment of both paternal and maternal DNA methylation imprinting, but it seems unnecessary for zygotic development [48, 53]. PHC1 and RNF2/RING1B are proteins belonging to the polycomb family involved in the regulation of chromatin structure through the modification of histones organisation [54]. Recent evidence suggests that the regulation of ESCs self-renewal or differentiation involves a balance between an Oct4, Nanog, Sox2 regulatory circuit, microRNAs and the polycomb repressive complexes. RING1B functions to ubiquitinate histone H2A and is implicated in the regulation of developmental genes [55]. In mouse ESCs, the proteins RING1A and RING1B are important in repressing developmental regulators and their binding to target genes is Oct4-dependent, suggesting a role downstream of the transcriptional regulatory network that maintains ESCs pluripotency [56, 57]. The dissociation of PHC1 and RING1B from the chromatin correlates with transcriptional silencing in mitosis and meiosis [58]. Furthermore, RING1B is important in early mouse development in the establishment of parental epigenetic asymmetry at constitutive heterochromatin and euchromatin until the late 8-cell stage, when this difference disappears and marks the end of the maternal-to-embryonic transition [59]. The importance of this protein in early development is also underscored by the arrest at gastrulation of *Ring1B*-null embryos [60].

*Gene cluster 5,* ‘Regulation of transcription’. The genes best known of this cluster are *Mta3* and *Zhx1*. The metastasis-associated MTA3 is involved in cancer cell migration by regulation of cell adhesion proteins [61]. The only gene of the Oct4-OETN group, *Zhx1*, is a member of zinc-finger and homeobox proteins that interacts with Dnmt3b *in vivo* and *in vitro* enhancing the transcriptional repression mediated by DNMT3B [62]. This cluster includes also *Gata1*, one of the 15 genes of the expanded Oct4-TN expressed exclusively in 2-cell embryos, known to function in mammalian erythropoiesis [63].

*Gene cluster 6*, ‘TGF Beta signalling’. This cluster includes four (*Fbxo15*, *Rbx1*, *Skp1a* and *Cul1*) Oct4-OETN genes. *Fbxo15*/*Fbx15* is an OCT4 target dispensable for ESCs self-renewal [64]. RBX1 (Ring box protein-1) is the Ring component of the Skp1-Cullin-F box protein ubiquitin ligase complex, whose disruption causes death at embryonic day 7.5 because of a failure in proliferation [65].

*Gene cluster 7*, ‘Anion exchanges’. This cluster contains one (*Scl4a8*) Oct4-OETN gene. These genes belong to a family of anion (HCO_3_^-^/Cl^-^) exchangers (AEs) active in mammalian cells to control their internal pH. This family of AEs is active in mouse oocytes, before and after ovulation [66] and in preimplantation embryos [67].

*Gene cluster 8*, ‘Apoptosis’. This cluster comprehends a group of nine (*Bag4*, *Bat3*, *Bcl2l12*, *Bcl2l13*, *Bcl2l2*, *Mcl1*, *Relt*, *1110007C09Rik* and *Prr18*) poorly known genes. The first seven have a pro-apoptotic or pro-survival function; whereas the function of the others is unknown. BAT3 assembles with BORIS and SEAT1a to exert effects on chromatin structure and gene expression [68].

*Gene cluster 9,* ‘Transcription factors-Cancer’. This cluster includes four (*Nfat5*, *Foxm1*, *Foxp1* and *Plagl1*) Oct4-OETN genes. Nfat5 (nuclear factor of activated T-cells 5) belongs to a family of transcription factors that are targets of plasma membrane integrin signalling pathways involved in promoting human carcinoma invasion [69]. *Foxm1* and *Foxp1* belong to the Forkhead-box (FOX) gene family. In humans, deregulation of FOX genes leads to congenital disorders, diabetes mellitus or carcinogenesis [70]. *Plagl1*/*Zac1* is an imprinted gene that encodes a zinc finger transcription factor inducing apoptosis and cell-cycle arrest, also its down-regulation is correlated with breast and ovary tumor or carcinomas [71-73]. Inactivation of maternally derived *Plagl1* leads to developmental malformations [74].

*Gene cluster 10*, ‘Copper distribution’. The three (*Atox1*, *Ccs* and *Commd1*) Oct4-OETN genes of this cluster are important in maintaining the correct copper distribution within the cell. Atox1 plays a role in copper homeostasis and is a breast cancer-associated protein whose dysregulation marks the pre-neoplastic stage [75]. Ccs is a copper chaperone for superoxide dismutase. *Commd1*/*U2af1-rs1* is an imprinted, paternally expressed gene, with an oocyte-specific methylation pattern [76, 77]. The methylation imprint of this gene, as well as that of three other imprinted genes (*Igf2r*, *Igf2* and *H19*), is lost upon derivation of ESCs from the ICM and is associated with aberrant imprinted gene expression in fetuses [78].

*Gene cluster 11*, ‘Translation-Cancer’. This cluster includes three (*Rps2*, *Rps20* and *Rps15*) Oct4-OETN genes. Of these three ribosomal proteins, RPS2 is the best known; it is over-expressed in malignant prostate cancer and is a target for *in vitro* therapeutic elimination of this tumor [79]; *Rps20* over-expression is associated with medulloblastoma, a malignant childhood brain tumor [80] and *Rps15* was found to be differentially expressed comparing normal liver tissues and liver cancer cell lines [81].

*Gene cluster 12*, ‘Subcortical maternal complex’. This cluster comprises four (*Filia*, *E330034G19Rik*, *Ooep*/*Floped* and *Tle6*) Oct4-OETN genes. Whilst the biological function of *E330034G19Rik* is still unknown, the function of the other three genes has been well characterised during oocyte growth and in early preimplantation development. FILIA, OOEP and TLE6 together with MATER are four maternal-effect proteins that interact together to make up for a sub-cortical maternal complex required for mouse preimplantation development beyond the 2-cell stage [82]. At the morula stage, FILIA and MATER proteins maintain their presence in the outer cells, whereas they disappear in the inner cells, providing a molecular marker of embryonic cell lineages [83].

*Gene cluster 13*, ‘GTPase-mediated signal transduction’, includes seven (*Rab14*, *Rab21*, *Rab31*, *Rab35*, *Rab39b*, *Gpsm3* and *AU040829*) Oct4-OETN genes. The Rab small GTPases, members of the RAS oncogene family, have been associated to cancer cell migration, tumor progression and invasiveness [84]. GPSM3 together with GPSM2 are members of the GoLoco family of proteins that regulate mitotic spindle orientation in cell division [85].

*Gene cluster 14*, ‘Signal transduction’, contains three (*Plekhm1*, *Rin3* and *AU040320*) poorly known Oct4-OETN genes. PLEKHM1 is involved in osteoclastic vesicular transport [87]; RIN3 plays a role in the transport pathway from the plasma membrane to the early endosomes [88].

*Gene cluster 15*, ‘Protein assembly’, encompasses four (*Polr2h*, *Parp16*, *Nsmce1* and *2310003F16Rik*) little know Oct4-OETN genes.

*Gene cluster 16*, ‘Protein phosphorylation’, comprises two (*Prkg1* and *Zc3h11a*) badly characterised Oct4-OETN genes. Interestingly, *Zc3h11a* contains and shares the same promoter with an intronless gene that encodes for a nuclear protein named Zbed6. Zbed6 regulates the expression of more than a 1000 genes involved in development, cell proliferation and growth, including the imprinted *Igf2* gene and members of the Sox and Fox families of transcription factors [89].

*Gene cluster 17*, ‘Regulation of transcription’. This cluster includes three (*Qars*, *Serf2* and *Smn1*) Oct4-OETN genes. Of these, only *Smn1* is well known because its homozygous deletion causes spinal muscular atrophy an autosomal recessive neuromuscular disorder [90].

*Gene cluster 18*, ‘Cell cycle regulation’. This cluster contains the highest number of Oct4-OETN genes (*Cdt1*, *Tipin*, *Stk24*, *2410091C18Rik*, *D10Ertd641e* and *2010107E04Rik*). Cdt1 is an important member of the origin recognition complex of DNA replication; over-expression of this gene may lead to malignant transformation [91]; Tipin is a timeless-interacting protein important in post-implantation development and is expressed in the adult brain [92].
