# Supplementary material for: Gatekeeper of pluripotency: A common Oct4 transcriptional network operates in mouse eggs and embryonic stem cells
Source: BMC Genomics. 2011 Jul 5;12:345. doi: 10.1186/1471-2164-12-345 (PMC3154874; doi:10.1186/1471-2164-12-345)
Supplement: Additional file 11 — RT-PCR primers. List of primers used for the real time RT-PCR analysis. [file 1471-2164-12-345-S11.DOC]

**Additional file 11.** RT-PCR primers.

| **Gene** | **Primer forward** | **Primer reverse** | **Amplicon length (bp)** |
| --- | --- | --- | --- |
| *Rps20* | 5’ CCATGGCATTTAAAGATACCG 3’ | 5’ AACCTTCTCCAGCGACTTCAC 3’ | 107 |
| *Prkg1* | 5’ TTGACACCTCCCATAATTCCA 3’ | 5’ CCTGAGTTGTCATCAGGTGGT 3’ | 104 |
| *Skp1a* | 5’ GGGACCAAGAATTCCTGAAAG 3’ | 5’ TGCATGTGACATCAAGCAAAC 3’ | 99 |
| *Mcl1* | 5’ AGCTTCATCGAACCATTAGCA 3’ | 5’ AAGAACTCCACAAACCCATCC 3’ | 104 |
| *Serf2* | 5’ AGATGATGGGCTTTCTGCTG 3’ | 5’ ACAAGGCTACTTGGGTTCCTC 3’ | 106 |
| *Oct4* | 5’ GTGGAGGAAGCCGACAACAATG 3’ | 5’ CACCTCACACGGTTCTCAATGC 3’ | 107 |
| *Htatsf1* | 5’ GAGAGACGAGCTGGACCAAA 3’ | 5’ TTCAGTACCAATGGGTCATCC 3’ | 101 |
